# Predicting surgical site infections after open reduction and internal fixation for ankle fractures

**DOI:** 10.1007/s00402-024-05623-9

**Published:** 2025-01-23

**Authors:** Jasper Tausendfreund, Diederick Penning, M. Azad Naryapragi, Kostan W. Reisinger, E. Tanis, P. Joosse, T. Schepers

**Affiliations:** 1 Present Address: Trauma Unit, Department of Surgery, Amsterdam UMC, University of Amsterdam, Meibergdreef 9, 1105 AZ, Amsterdam, the Netherlands; 2 Present Address: Department of Trauma Surgery, Noordwest Clinics, Wilhelminalaan 12, 1815 JD, Alkmaar, the Netherlands; 3 Present Address: Trauma Unit, Department of Surgery, Amsterdam UMC, location VUmc, De Boelelaan 1117, 1081 HV, Amsterdam, the Netherlands

**Keywords:** Ankle fracture, Fractures, Risk factors, Surgical site infection, Fracture related infection

## Abstract

**Introduction:**

Surgical site infections (SSI) are one of the more severe complications following ankle surgery. It is associated with worse outcomes and re-admissions. Therefore, identification of risk factors is essential. The aim of this study was to identify risk factors for SSI in patients undergoing surgery for ankle fractures.

**Materials and methods:**

A retrospective study was performed in a large cohort (*n* = 929) of patients who underwent open reduction and internal fixation (ORIF) of ankle fractures between 2015 and 2020 in the Netherlands. The primary outcome variables included rate of SSI (superficial or deep) and deep SSI. Prediction factors were categorized as patient-related, injury-related and treatment-related.

**Results:**

The incidence rate was 9.36% for SSI and 3.55% for deep SSI. Univariate analysis showed significant associations for higher age (*p *< 0.001), DM (*p* = 0.018), ASA 2 and 3 (*p* = 0.013 and *p *< 0.001), bi- and trimalleolar fractures (*p* = 0.021 and *p* = 0.013), open fractures (*p* = 0.004) and small size plate compared to screw fixation (*p* = 0.027). The only independent significant risk factor for SSI in multivariate analysis was open fracture. For deep SSI the significant risk factors were DM (*p* = 0.039), ASA 3 and 4 (*p* = 0.001 and *p* = 0.005) and open fracture (*p* = 0.002). After multivariate analysis, the independent significant risk factors were open fracture and ASA 3 and 4.

**Conclusions:**

Higher age, DM, ASA 2 and 3, bi- and trimalleolar fractures, open fractures and standard plate-size implant placement were identified as significant risk factors for SSI. Open fracture was the only significant independent risk factor for SSI after ORIF of ankle fractures. In deep SSI, there were different risk factors. DM, ASA 3 and 4, and open fractures were significantly associated. Although, open fracture and ASA 3 and 4 were the significant independent risk factors.

## Introduction

 A surgical stabilization is required in approximately 50% of all ankle fractures [1, 2]. One of the more severe complications of ankle surgery is a surgical site infection (SSI). This has an average incidence of 7.19% in the general population according to Shao et al. and 9–10% in other studies [3–9]. In certain risk populations, such as elderly or patients with dislocated or open fracture, incidence rates up to 17% are reported [10–12]. Deep SSI usually require surgical intervention and these rates vary between 2.8–6.8% [13, 14].

SSI are associated with an increased risk of joint dysfunction, delayed union, malunion and in severe cases even ankle arthrodesis or amputation [11, 15]. Furthermore, it could lead to prolonged hospitalization or re-admissions [9, 13, 16] . Treatment of deep SSI usually includes long-term antibiotic treatment and in some cases re-operation [15]. SSI’s are associated with an increase in costs and therefore, the prevention of SSI decreases overall healthcare costs [17–19].Therefore, to potentially reduce the risk of SSI, identification of risk factors is essential [11, 15, 16].

In previous studies, multiple risk factors have been identified [6, 20]. One meta-analysis showed significantly increased risk of infection for patients with an American Society of Anesthesiologists score (ASA) ≥ 3, higher body mass index (BMI), chronic heart disease, open fracture, severe fracture dislocation, wound contamination grade 2–4 and absence of antibiotic prophylaxis [9]. Smaller studies demonstrated various other risk factors for infection such as higher age, diabetes mellitus (DM), male sex, trimalleolar fractures, experience of the surgeon, duration of surgery of more than 90 min, immunosuppressive medication and tourniquet use [6–8, 11, 21, 22] .

The aim of this study was to identify which patient, trauma and treatment-related factors result in an increased risk of infection in ankle fractures that required open reduction internal fixation (ORIF).

## Methods

This study retrospectively reviewed the records of all patients with an ankle fracture who were surgically treated between January 2015 and December 2020. Patients from two large non-academic hospitals and one academic medical centre in the Netherlands were included. Patients were selected from the electronic patient file (EPF) using procedural and diagnostic codes. Data was extracted by two authors and a consensus meeting was scheduled in case of any discrepancies.

**Fig. 1:**
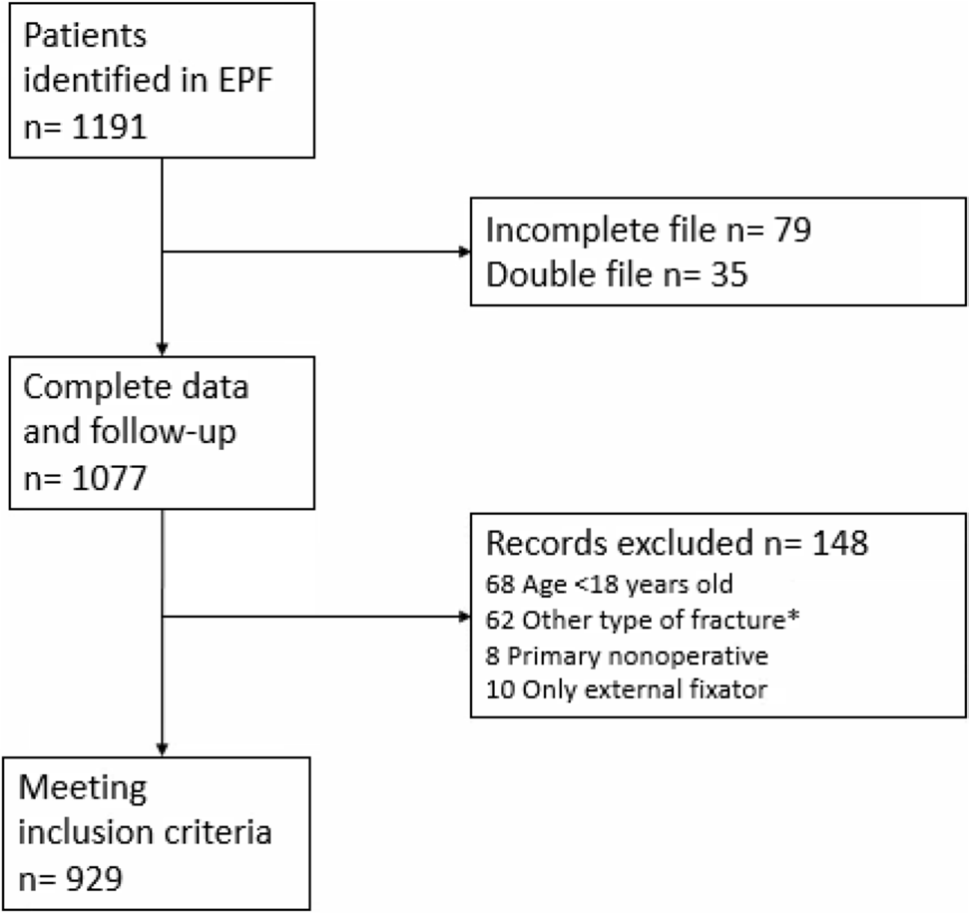
Flow diagram of patient selection *Other type of fracture: calcaneus, distal tibia, cruris, metatarsal, talus

Inclusion criteria were age ≥ 18 years, surgical treatment of an ankle fractures with open reduction and internal fixation, minimal follow-up of 12 months. Patient selection is shown in Fig. 1.

Patient characteristics included gender, age, weight, BMI, DM, smoking status, alcohol abuse (> 2 units/day) and ASA classification.

Injury-related variables included fracture type classified using Weber, Lauge Hansen and the number of malleoli involved, which has also been described as Pott’s classification [23, 24]. Fracture of the posterior malleolus was registered separately. Open fractures were classified according to the Gustilo classification [25]. Fracture dislocation was classified as > 50% tibio-talar dislocation on the X-ray or when a closed reduction by the ambulance or at the emergency department was performed in case of clinically severe dislocation.

Treatment-related variables included date of surgery, time from injury to ORIF, reduction, use of cast, time from injury to external fixation, time from external fixation to ORIF and plate size (small with 3.5 mm screws vs. mini with 2.7 mm screws vs. screws only). The choice for fixation material depended on the surgeon and the type of fracture.

Outcome variables included SSI, classified as superficial or deep by the treating clinician according to the CDC criteria. Patients with redness as isolated symptom, without pus or wound fluid, that were treated with short or long course antibiotics, were also included. We consecutively performed a separate analysis on the deep SSI.

This study was assessed by the local medical ethics testing committees for IRB approval. Patients from the Academic medical centre were asked to sign a no-objection statement according to the METC requirements (W21_074 # 21.084). Patients from the two non-academic hospitals did not need to be asked for a no-objection statement, according to the assessment of their local METC.

### Statistical analysis

Statistical analysis was performed using IBM Statistical Package for Social Sciences (SPSS) 26.0.0.1.

For statistical data analysis Pearson Chi-square, and Likelihood ratio were used to compare the categorical variables. Continuous variables were tested for normality with the Kolmogorov-Smirnov and Shapiro-Wilk tests. Non-normal distributed variables were compared using the Mann-Whitney U test and displayed as median with interquartile range (IQR). Normal distributed variables were compared using a t-test and displayed with a mean and standard deviation (SD).

Binary logistic regression was used for univariate and multivariate analyses to calculate if the outcome variables were significantly dependent to demographic, injury-related of treatment-related variables. Univariate analysis was used to identify which single variables were significant risk factors. The significant risk factors from the univariate analysis were then further examined in the multivariate analysis to correct for confounders. This allowed us to identify which risk factors are independently associated with SSI.

Odds ratios (OR) with 95% confidence interval (CI) were also calculated using binary logistic regression.

For all statistical tests, a p-value of < 0.05 was considered statistically significant.

### Results

A total of 929 patients with ORIF for ankle fractures were included and 87 of these patients developed SSI (9.36%). Table 1 shows the demographics of the cohort. Deep SSI presented in 33 patients (3.55%).

**Table 1:** Demographic data of all patients with ORIF for ankle fracture

	No SSI *n* = 842	SSI *n* = 87	Total *n* = 929
Gender Male Female	842 (100%) 351 (41.7%) 491 (58.3%)	87 (100%) 38 (43.7%) 49 (56.3%)	929 (100%) 389 (41.9%) 540 (58.1%)
Age (median in years, [IQR])	50.0 [31]	57.0 [28]	51.0 [30]
BMI (median, [IQR])	26.6 [6]	27.2 [7]	26.6 [6]
Time to ORIF (median in days, [IQR])	7.0 [10]	6.0 [10]	7.0 [10]
Diabetes mellitus Yes No	840 (100%) 63 (7.5%) 777 (92.5%)	87 (100%) 13 (14.9%) 74 (85.1%)	927 (100%) 76 (8.2%) 851 (91.8%)
Smoking Yes No	663 (100%) 182 (27.5%) 481 (72.5%)	68 (100%) 20 (29.4%) 48 (70.6%)	731 (100%) 202 (27.6%) 529 (72.4%)
Alcohol abuse Yes No	647 (100%) 90 (13.9%) 557 (86.1%)	67 (100%) 10 (14.9%) 57 (85.1%)	714 (100%) 100 (14.0%) 614 (86.0%)
ASA-score 1 2 3 4	839 (100%) 308 (36.7%) 421 (50.2%) 101 (12.0%) 9 (1.1%)	87 (100%) 17 (19.5%) 48 (55.2%) 20 (23.0%) 2 (2.3%)	926 (100%) 325 (35.1%) 469 (50.6%) 121 (13.1%) 11 (1.2%)
Malleoli Unimalleolar Bimalleolar Trimalleolar	840 (100%) 201 (23.9%) 261 (31.3%) 378 (45.0%)	87 (100%) 10 (11.5%) 31 (35.6%) 46 (52.9%)	927 (100%) 211 (22.8%) 292 (31.5%) 424 (45.7%)
Posterior malleolar fracture Yes No	842 (100%) 481 (57.1%) 361 (42.9%)	87 (100%) 56 (64.4%) 31 (35.6%)	929 (100%) 537 (57.8%) 392 (42.2%)
Weber Not applicable A B C	829 (100%) 35 (4.2%) 12 (1.4%) 537 (64.8%) 245 (29.6%)	87 (100%) 1 (1.1%) 0 (0.0%) 58 (66.7%) 28 (32.2%)	916 (100%) 36 (3.9%) 12 (1.3%) 595 (65.0%) 273 (29.8%)
Lauge-Hansen Not applicable Supination Adduction Supination External rotation Pronation External Rotation Pronation Abduction	825 (100%) 19 (2.3%) 11 (1.3%) 536 (65.0%) 234 (28.4%) 25 (3.0%)	87 (100%) 2 (2.3%) 0 (0.0%) 58 (66.7%) 26 (29.9%) 1 (1.1%)	912 (100%) 21 (2.3%) 11 (1.2%) 594 (65.1%) 260 (28.5%) 26 (2.9%)
Dislocation Yes No	842 (100%) 466 (55.3%) 376 (44.7%)	87 (100%) 54 (62.1%) 33 (37.9%)	929 (100%) 520 (56.0%) 409 (44.0%)
Open fracture Yes No	842 (100%) 66 (7.8%) 776 (92.2%)	87 (100%) 15 (17.2%) 72 (82.8%)	929 (100%) 81 (8.7%) 848 (91.3%)
Gustilo classification I II III	66 (100%) 25 (37.9%) 24 (36.4%) 17 (25.8%)	15 (100%) 5 (33.3%) 7 (46.7%) 3 (20.0%)	81 (100%) 30 (37.0%) 31 (38.3%) 3 (24.7%)
Cast Yes No	842 (100%) 625 (74.2%) 217 (25.8%)	87 (100%) 68 (78.2%) 19 (22.1%)	929 (100%) 693 (74.6%) 236 (25.4%)
External Fixator Yes No	842 (100%) 53 (6.3%) 789 (93.7%)	87 (100%) 7 (8.0%) 80 (92.0%)	929 (100%) 60 (6.5%) 869 (93.5%)
Type of implant Screw Mini size plate 2.4/ 2.7 mm Small size plate 3.5 mm	842 (100%) 112 (13.3%) 44 (5.2%) 686 (81.5%)	87 (100%) 4 (4.6%) 5 (5.7%) 78 (89.7%)	929 (100%) 116 (12.5%) 49 (5.3%) 764 (82.2%)

Table 2 shows the univariate analysis of the variables on SSI. Significant correlations were seen for higher age (*p *< 0.001), DM (*p* = 0.018), ASA 2 and 3 (*p* = 0.013 and *p *< 0.001), bi- and trimalleolar fractures (*p* = 0.021 and *p* = 0.013), open fractures (*p* = 0.004) and use of small size plate versus only screw fixation (*p* = 0.027).

**Table 2:** Univariate analysis SSI

Factor	OR (95% CI)	*P*-value
Gender	0.922 (0.591–1.439)	0.72
Age	1.023 (1.011–1.036)	**< **0.001
BMI	1.034 (0.986–1.084)	0.167
Time to ORIF	0.985 (0.946–1.025)	0.445
Diabetes mellitus	2.167 (1.139–4.121)	0.018
Smoking	1.101 (0.636–1.906)	0.731
Alcohol abuse	1.086 (0.535–2.204)	0.82
ASA		
* 1*	Ref.	
* 2* * 3* * 4*	2.066 (1.165–3.661) 3.588 (1.809–7.114) 4.026 (0.806–20.10)	0.013 < 0.001 0.09
Malleoli		
* Unimalleolar*	Ref.	
* Bimalleolar* * Trimalleolar*	2.387 (1.143–4.984) 2.446 (1.209–4.950)	0.021 0.013
Posterior malleolar fracture	1.356 (0.856–2.147)	0.194
Weber		
* Not applicable*	Ref.	
* A* * B* * C*	0.000 3.780 (0.508–28.11) 4.000 (0.528–30.33)	0.999 0.194 0.180
Lauge-Hansen		
* Not applicable*	Ref.	
* Supination-adduction* * Supination-external rotation* * Pronation-external rotation* * Pronation-abduction*	0.000 1.028 (0.234–4.525) 1.056 (0.223–4.789) 0.380 (0.032–4.508)	0.999 0.971 0.994 0.443
Fracture dislocation	1.320 (0.839–2.079)	0.23
Open fracture	2.449 (1.330–4.510)	0.004
Gustilo		
* I*	Ref.	
* II* * III*	1.458 (0.407–5.230) 0.882 (0.186–4.192)	0.563 0.875
Cast	1.243 (0.730–2.114)	0.423
External fixator	1.301 (0.572–2.957)	0.530
Type of implant		
* Screw*	Ref.	
* Mini size plate 2.4/ 2.7 mm* * Small size plate 3.5 mm*	3.182 (0.816–12.40) 3.184 (1.143–8.868)	0.095 0.027

The significant variables of the univariate analysis were included in a multivariate logistic regression analysis which is shown in Table 3. In the multivariate analysis, open fractures were identified as risk factor for the development of SSI (*p* = 0.018). A number of significant variables in the univariate analysis (age, DM, ASA classification, number of involved malleoli and type of implant) were not significant in the multivariate analysis.

**Table 3:** Multivariate analysis SSI

Factor	OR (95% CI)	*P*-value
Age	1.011 (0.997–1.026)	0.135
Diabetes Mellitus	1.324 (0.656–2.672)	0.433
ASA		0.247
Uni-/bi-/trimalleolar		0.389
Open fracture	2.146 (1.141–4.034)	0.018
Type of implant		0.179

In Table 4, the univariate analysis of the variables on deep SSI. DM (*p* = 0.039), ASA 3 and 4 (*p* = 0.001 and *p* = 0.005) and open fracture (*p* = 0.002) were significantly correlated with deep SSI.

**Table 4:** Univariate analysis deep SSI

Factor	OR (95% CI)	*P*-value
Gender	0.860 (0.428–1.729)	0.673
Age	1.005 (0.986–1.024)	0.610
BMI	1.032 (0.962–1.107)	0.385
Time to ORIF	0.972 (0.912–1.036)	0.385
Diabetes mellitus	2.622 (1.047–6.564)	0.039
Smoking	1.103 (0.475–2.562)	0.819
Alcohol abuse	0.482 (0.112–2.069)	0.327
ASA		
* 1*	Ref.	
* 2* * 3* * 4*	1.637 (0.623–4.306) 5.333 (1.927–14.761) 11.852 (2.097–66.986)	0.318 0.001 0.005
Malleoli		
* Unimalleolar*	Ref.	
* Bimalleolar* * Trimalleolar*	2.550 (0.926–7.024) 1.103 (0.378–3.215)	0.070 0.858
Posterior malleolar fracture	0.598 (0.297–1.201)	0.148
Weber		
* Not applicable*	Ref.	
* A* * B* * C*	0.000 1.469 (0.193–11.174) 1.053 (0.128–8.669)	0.999 0.711 0.962
Lauge-Hansen		
* Not applicable*	Ref.	
* Supination-adduction* * Supination-external rotation* * Pronation-external rotation* * Pronation-abduction*	0.000 0.868 (0.841–0.108) 0.551 (0.065–4.704) 0.800 (0.047–13.604)	0.999 0.868 0.586 0.877
Fracture dislocation	1.071 (0.530–2.162)	0.849
Open fracture	3.616 (1.575–8.305)	0.002
Gustilo		
* I*	Ref.	
* II* * III*	1.500 (0.233–9.677) 2.471 (0.374–16.320)	0.670 0.348
Cast	1.271 (0.545–2.969)	0.579
External fixator	0.926 (0.218–3.996)	0.926
Type of implant		
* Screw*	Ref.	
* Mini size plate 2.4/ 2.7 mm* * Small size plate 3.5 mm*	7.500 (0.760–73.997) 4.525 (0.610–33.541)	0.084 0.140

The significant variables in Table 4 were included in the multivariate logistic regression analysis which is shown in Table 5. Only open fracture and ASA 3 and 4 were significant independent variables for deep SSI, as DM was not significant in multivariate analysis.

**Table 5:** Multivariate analysis deep SSI

Factor	OR (95% CI)	*P*-value
Diabetes Mellitus	1.424 (0.518–3.915)	0.494
ASA		
* 1*	Ref.	
* 2* * 3* * 4*	1.523 (0.576–4.027) 4.950 (1.776–13.792) 8.884 (1.495–52.787)	0.396 0.002 0.016
Open fracture	3.149 (1.335–7.430)	0.009

### Discussion

In this retrospective multicenter study, all ankle fractures receiving ORIF were included. The aim of the study was to identify which variables could predict a higher risk for a surgical site infection after surgical treatment for ankle fractures. After univariate analysis, significant risk factors were age, DM, higher ASA, bi- and trimalleolar fractures, open fractures and the use of standard small size plate compared to screws only. After multivariate analysis, open fractures were found to be the only significant independent predictive factor for SSI.

To our knowledge, this study is one of the largest multicenter retrospective studies in Western Europe on prediction factors for SSI including a wide variety of patient-related, injury-related and treatment-related variables, with a long-term follow-up of at least 1 year.

Univariate analysis presented age, DM, ASA 2 and 3, bi- and trimalleolar fractures, open fractures and the use of a small-fragmente plate (versus screws only) as significant univariate prediction factors for SSI. Similar results have been found in other studies. In the studies of Sun et al. and Sanders et al., age was found to be a significant risk factor for wound complications, respectively univariate and multivariate [22]. However, they included different types of surgery and multiple trauma related surgeries of the lower extremity. A meta-analysis of Shao et al. has also shown DM is a significant risk factor for infection in ankle surgery [8]. Similar to our results they also described ASA ≥ 3 as a significant risk factor [8]. Trimalleolar fractures was found to be an significant predictor for infection by Sato et al [7]. Smoking is a frequently described risk factor for infection. In our study, smoking is not a significant risk factor.

Schepers et al., compared one-third tubular plate to a locking compression plate, the results suggested that the locking compression plate showed a significant increase in infection [26]. And in an other study, mini-fragment plates had a SSI rate of 3.1% and small-size plates 9.1%, which was not statistically significant (*p* = 0.161) [27]. In the current study, the thickness of the plates was not accounted for. Screws only were less likely to have wound complications compared to plate fixation.

Fibula nails were not included in this study, although literature suggests the use of the fibula nail leads to significantly less wound complications, there is no difference in total complications compared to ORIF [28].

These univariate results should be interpreted with care as there is probably a selection bias, as patients and fracture patterns that allow for screw only fixation are probably younger with high quality bone and had low energy/non-comminuted fractures, but this study did not investigate this.

Significant prediction factors in other studies, which were not included in this study, were chronic heart disease (in the older population), high energy injury, skill level of the surgeon, surgery time and tourniquet use during the operation [8, 14, 21, 22] .

Benedick et al. (2020), studied the effect of tourniquet use on infectious complications in ankle fractures [29]. It showed that tourniquet use does not affect the rates of infections or wound healing problems.

Post-operative malreduction was not included in our variables. Some studies showed post-operative malreduction as a significant risk factor for infection [14, 30].

Gender, severe fracture dislocation and higher body mass index were significant risk factors in other studies, but no significant difference was found [6–9, 22, 31]. Various studies had open fracture as independent risk factor for infection [8, 21, 22, 32].

Although time to ORIF was not a significant risk factor in our population, literature suggests that delayed surgery is a risk factor for infectious complications. Surgery should preferably be performed within the first day [33].

The strengths of this study are the multicenter character, which provides more generalizability and the relatively high number of patients included with a wide variety of risk factors and at least a one-year follow-up. The retrospective design is one of the limitations, as it is more susceptible for selection bias. In some cases, patient information is missing on medical history, smoking status or physical examination.

This study included all ankles which received surgical treatment. Although univariate analysis showed DM, higher ASA, bi- and trimalleolar fractures, and the use of standard small size plate compared to screws only as significant risk factors, these could not be independently associated as risk factors for SSI. Only the open fractures were a significant independent risk factor for SSI, and open fracture, ASA 3 and 4 for deep SSI.

In most patients with an open ankle fracture, antibiotic prophylaxis would be administered at presentation [34]. Despite this, a lot of patients with open fractures develop a SSI (17.2%). Literature suggests that antibiotic prophylaxis should be administered within 3 h of injury [35]. In case of deep SSI, the Gold Standard for treatment is surgical debridement and deep tissue culture sampling. After surgery long term antibiotic treatment when the osteosynthesis material is still in situ [36, 37]

### Conclusion

The cohort in this study had 9.36% SSI after ankle surgery. Univariate analysis showed the significant risk factors were higher age, DM, ASA 2 and 3, bi- and trimalleolar fractures, open fractures and standard small-fragment plate fixation. Multivariate analysis however showed that only an open fracture is a significant independent risk factor for SSI after ORIF of ankle fractures. Univariate analysis of a subset of patients with deep SSI showed DM, ASA 3 and 4, and open fractures were significant risk factors, but in multivariate analysis only open fractures and ASA 3 and 4 remained significant.
